# A Computational Analysis of Aberrant Delay Discounting in Psychiatric Disorders

**DOI:** 10.3389/fpsyg.2015.01948

**Published:** 2016-01-13

**Authors:** Giles W. Story, Michael Moutoussis, Raymond J. Dolan

**Affiliations:** ^1^Max Planck University College London Centre for Computational Psychiatry and Ageing Research, University College LondonLondon, UK; ^2^Wellcome Trust Centre for Neuroimaging, University College LondonLondon, UK; ^3^Centre for Health Policy, Imperial College London, Institute of Global Health Innovation, St. Mary's HospitalLondon, UK

**Keywords:** discounting, time preference, psychiatric, computational psychiatry, mental illness, biopsychosocial

## Abstract

Impatience for reward is a facet of many psychiatric disorders. We draw attention to a growing literature finding greater discounting of delayed reward, an important aspect of impatience, across a range of psychiatric disorders. We propose these findings are best understood by considering the goals and motivation for discounting future reward. We characterize these as arising from either the opportunity costs of waiting or the uncertainty associated with delayed reward. We link specific instances of higher discounting in psychiatric disorder to heightened subjective estimates of either of these factors. We propose these costs are learned and represented based either on a flexible cognitive model of the world, an accumulation of previous experience, or through evolutionary specification. Any of these can be considered suboptimal for the individual if the resulting behavior results in impairments in personal and social functioning and/or in distress. By considering the neurochemical and neuroanatomical implementation of these processes, we illustrate how this approach can in principle unite social, psychological and biological conceptions of impulsive choice.

## Introduction

Vitae summa brevis spem nos vetat incohare longam

Life's short span forbids our embracing far-reaching hopes - Horace, Odes (23BC)

Humans and animals often accept a smaller reward immediately, rather than wait to receive a larger reward in the future (Ainslie, 1974; Thaler, 1981; Thaler and Shefrin, 1981; Fishburn and Rubinstein, 1982; Frederick et al., 2002; McClure et al., 2007; Kalenscher and Pennartz, 2008; Pine et al., 2009). In economic terms, this behavior indicates that the subjective value of reward decreases as it is delayed, a process referred to as temporal discounting (for reviews see Frederick et al., 2002; Kalenscher and Pennartz, 2008). As we will discuss, biological agents have good reason to discount delayed rewards, since these might either fail to materialize or arrive too late to satisfy the organism's current needs. Indeed, as pointed out by the Roman poet Horace in the quotation above, the ultimate motive for discounting is that the agent will die before deferred rewards are realized.

In humans, temporal discounting can be measured by examining choices between quantities of money at varying delays (Mazur, 1987; Kirby and Maraković, 1995; Myerson et al., 2001; Green and Myerson, 2004). The most commonly used method elicits choices between a larger, delayed amount of money, (e.g., “$100 in 6 months”), and a series of immediate amounts of decreasing magnitude (e.g., “$80 today”). By observing at each delay the magnitude of smaller-sooner reward at which the participant switches to preferring the later reward, the decrease in value of the later reward can be plotted as a function of delay. A non-parametric estimate of discounting can be derived by taking the area beneath this indifference curve (Myerson et al., 2001). Alternatively, the shape of the curve can be fitted with a discount *function*.

Samuelson (1937), and later Strotz (1957), showed that a decision-maker who discounts future benefits according to an exponentially decreasing function (and behaves *as if* to maximize the sum of exponentially discounted reward) allocates resources across time in a self-consistent manner. Under the classical model, the effect of delay, *d*, is described by an (exponential) discount function, here denoted byΔ(*d*), such that:

(1) $$\begin{array}{l} △\left(d\right)\;=\;e^{-kd} \end{array}$$

Where *k* is an exponential discount rate, such that higher values of *k* lead to a steeper decrease in reward value with delay. The effect of reward magnitude, here signified by *r*, is independently described by an instantaneous utility function, *u*(*r*), such that the subjective utility of a stream of future rewards is then given by:

(2) $$\begin{array}{l} U\left(r_{t},r_{t+1},r_{t+2}\ldots r_{T-1},r_{T}\right)\;=\;\overset{T}{\underset{t}{\sum }}u\left(r_{\tau }\right)△\left(\tau -t\right) \end{array}$$

As reviewed by Frederick et al. (2002), the above account was not intended as a veridical psychological model of choice over time. In keeping with this, many experimental studies have shown that a discounting function is better approximated via a hyperbolic than an exponential function (e.g., Green et al., 1994; Kirby and Herrnstein, 1995; Kirby and Maraković, 1995; Myerson and Green, 1995; Laibson, 1997; van der Pol and Cairns, 2002; Rubinstein, 2003), of the form:

(3) $$\begin{array}{l} \;\Delta \left(d\right)=\frac{1}{1+kd} \end{array}$$

Here *k* denotes a hyperbolic discount rate (though for alternative accounts see Read, 2001; Kable and Glimcher, 2010; Read et al., 2012; Luhmann, 2013).

Temporal discounting has received considerable attention in human behavioral neuroscience, not least because many forms of maladaptive behavior are readily characterized as pursuit of immediate gratification at the expense of reaping greater rewards in the future (Critchfield and Kollins, 2001; Bickel et al., 2007, 2014a; Koffarnus et al., 2013; Story et al., 2014). Indeed, lending validity to the discounting construct, steeper discounting is positively associated with behaviors with potentially harmful long-term consequences such as tobacco smoking (Odum et al., 2002; Epstein et al., 2003; Reynolds et al., 2004; Bickel et al., 2008; MacKillop and Kahler, 2009; Fields et al., 2009a,b; Reynolds and Fields, 2012), alcohol use (Van Oers et al., 1999; Mazas et al., 2000; Petry, 2001; Field et al., 2007; Reynolds et al., 2007; Rossow, 2008; MacKillop and Kahler, 2009; Moore and Cusens, 2010), illicit drug misuse (Kirby et al., 1999; Petry and Casarella, 1999; Kollins, 2003; Petry, 2003; Kirby and Petry, 2004; Washio et al., 2011; Stanger et al., 2012), credit card debt (Meier and Sprenger, 2012) and risky sexual or drug-taking practices (Odum et al., 2000; Dierst-Davies et al., 2011). Also, many authors have explored how discounting relates to demographic variables, finding that measured discounting decreases across the lifespan (Green et al., 1996, 1999; Chao et al., 2009; Steinberg et al., 2009), is negatively correlated with income (Green et al., 1996; Eckel et al., 2005; Reimers et al., 2009), and tends to be lower in individuals living in the developed world than in the developing world (Wang et al., 2010). Furthermore, although discounting is sensitive to a gamut of contextual factors (for a review see Koffarnus et al., 2013), the level of discounting has been shown to exhibit high test-retest reliability when measured under similar conditions (Odum, 2011), and the extent of individual discounting for different forms of reward is correlated (Odum, 2011), suggesting that discounting has a substantial trait component.

More recently, researchers have taken an interest in comparing discounting behavior in groups who exhibit symptoms of a given psychiatric disorder and those who do not. These studies have found evidence for steeper discounting amongst patients with symptoms of schizophrenia (Heerey et al., 2007, 2011; Ahn et al., 2011; MacKillop and Tidey, 2011; Wing et al., 2012; Avsar et al., 2013; Weller et al., 2014), depression (Takahashi et al., 2008; Dennhardt and Murphy, 2011; Dombrovski et al., 2012; Imhoff et al., 2014; Pulcu et al., 2014), mania (Mason et al., 2012), attention deficit hyperactivity disorder (ADHD) (Barkley et al., 2001; Tripp and Alsop, 2001; Bitsakou et al., 2009; Paloyelis et al., 2010a,b; Scheres et al., 2010; Scheres and Hamaker, 2010), anxiety disorder (Rounds et al., 2007) and cluster B personality disorder (Dougherty et al., 1999; Moeller et al., 2002; Petry, 2002; Dom et al., 2006a,b; Lawrence et al., 2010; Coffey et al., 2011). This line of enquiry is not without theoretical justification, for example the broader construct of impulsivity, defined as taking action without forethought or regard for consequences (Moeller et al., 2001), of which discounting is an element, is a defining feature of some psychiatric disorders, for example borderline personality disorder (Moeller et al., 2001; DSM V, 2013) and mania (Swann, 2009). Also, psychiatric disorders are strongly associated with poor health choices, including but not limited to cigarette smoking, and drug and alcohol misuse (Robson and Gray, 2007), which have themselves been associated with steeper discounting (Bickel et al., 2012b, 2014a,b; Story et al., 2014). However, in many cases this research, although clearly valuable, appears to have been opportunist.

In this article we attempt to understand increases in discounting seen across a range of psychiatric disorders in light of the *reasons* why people should discount the future in the first place. We propose that the study of intertemporal impulsivity in psychiatric disorders would benefit from fractionating these underlying motives, and that parsing discounting in this manner can assist in drawing out the contributing psychological and biological processes. Our approach follows that of the neuroscientist David Marr (Marr, 1982), who proposed that information processing systems can be understood at three levels of analysis: a “computational” level, specifying what information processing problem is being solved by the system, an “algorithmic” level, formalizing how the system attempts to solve the problem, and an “implementational” level, denoting how these processes are realized physically.

For the case of discounting, the computational problem is easily defined in economic terms: to optimize the sum of future reward. However, this definition obscures a difficult question as to what constitutes “reward” (Moutoussis et al., 2015). It is convenient here to assume that all biological agents share some fundamental objective function. Rather than attempting to characterize the objective function directly, we assume some consensus on the kinds of outcome that organisms often seek, and that can therefore be considered “rewarding.” We then consider a subset of generic scenarios under which behavior consistent with discounting would indeed optimize the sum of future “reward.” This will give us some insight as to the contexts that agents, who discount future reward in different ways, including humans deemed to have mental disorders, might be adapted to.

We go on to speculate as to the broad classes of algorithms that biological agents might use to optimize reward, and where relevant their possible neural implementation. We argue that the application of this approach to psychiatric disorders, the bedrock of the emerging field of computational psychiatry (Huys et al., 2011; Montague et al., 2012; Friston et al., 2014; Stephan and Mathys, 2014; Wang and Krystal, 2014), can help to bridge a gap between psychological and biological conceptions of mental ill health (for further discussion see Moutoussis et al., 2015).

## Marr's computational level: reasons to discount future reward

The discount function estimated from the analysis of intertemporal choice paradigms is likely to reflect the influence of factors jointly serving to make impatience potentially advantageous. A key ambiguity in the classical economic model concerns whether these factors should be properly assigned to the time series of future rewards, or to the discount function (Frederick et al., 2002; Frederick and Loewenstein, 2008; Friston et al., 2013; for a review of contextual influences on discounting see Koffarnus et al., 2013). The following discussion illustrates that if they are made fully explicit in the utility function, behavior consistent with temporal discounting emerges.

### Opportunity cost

#### Growth and missed investment

For most organisms growth and development are necessary to reach reproductive capacity (Williams, 1957). For humans, development also extends to furthering one's social status. Growth potential motivates obtaining rewards sooner rather later, since earlier rewards can be invested—effectively loaned out at some rate of interest (see Rachlin, 2006; Kacelnik, 2011). The form of discounting that results depends on whether or not interest can be re-invested. Under the most straighforward scenario, referred to as simple interest, interest is not reinvested during the term of the loan. Consider a reward with utility *r* (for simplicity we omit the instantaneous utility function) invested for a period of time, *d*, to yield a larger payout, *R*. With simple interest:

(4) $$\begin{array}{l} R\;=\;r\;+\;krd \end{array}$$

Solving for *r* and expressing as a ratio of the payout gives:

(5) $$\begin{array}{l} \frac{r}{R}\;=\;\frac{1}{1\;+\;kd} \end{array}$$

A decision-maker should therefore be indifferent between a larger reward of utility, *R*, received after a delay, *d*, and a smaller reward, *r*, received immediately. Thus, linear growth (simple interest) motivates hyperbolic discounting (see Read, 2004; Rachlin, 2006).

In the above example, after the delay has lapsed the agent ought to reclaim their money and re-invest the entire payout to avoid losing out to a lower rate of interest. Compound interest represents a continual reinvestment of the payout, and generates exponential growth, such that the payout accrued at time *d* after choosing *r* is given by:

(6) $$\begin{array}{l} R\;=\;re^{gd} \end{array}$$

Where *g* reflects the interest rate. Rearranging as before gives:

(7) $$\begin{array}{l} \frac{r}{R}\;=\;e^{-gd} \end{array}$$

Thus, compound interest motivates exponential discounting.

#### Missed income

In the natural world, delay often entails inactive waiting, during which other sources of reward cannot be harvested. The cost associated with an inactive delay can be quantified as the reward that is missed out on while waiting (Kacelnik, 2011). Under one such formulation, organisms should consequently choose an action which maximizes a *rate* of reward per unit time, a concept that has arisen in ecological theory independently from the notion of discounting (Stevens and Krebs, 1986). Under this formulation, discounted value is simply inversely proportional to delay (Chung and Herrnstein, 1967). It can be easily shown however that if even “immediate” rewards are associated with some small delay, *m*, where *m* = 1∕*k*, this is equivalent to hyperbolic discounting (Daw and Touretzky, 2000). Thus, at indifference:

(8) $$\begin{array}{l} \frac{r}{m}\;=\;\frac{R}{m\;+\;d} \end{array}$$

Rearranging as previously:

(9) $$\begin{array}{l} \frac{r}{R}=\frac{m}{m+d}=\frac{1}{1+d∕m}=\frac{1}{1+kd} \end{array}$$

A corollary of this theory is that the opportunity cost of delaying reward on a particular option depends on the average rate of reward from all other options (Chung and Herrnstein, 1967; Daw and Touretzky, 2000; Niv et al., 2007).

Inactive waiting leads to interesting results if other options become available only once the delays associated with the current choice have lapsed. Consider for example a lawyer who is paid by the hour for seeing clients at weekdays, but does not work at weekends. Say that he or she has two lunch options, either waiting in a long queue for a tasty lunch at a popular café, or being able to buy an equally calorific but less enjoyable meal straightaway at a sandwich bar. The lawyer might be optimally inclined to choose the sandwich bar on weekdays, so as to facilitate a sooner return to work, but might choose to wait at the café if faced with the same choice on a weekend. Here the intertemporal choice is influenced by other available sources of reward, which are inaccessible during the delay. In ecological terms, if an organism is foraging in a reward-rich area, the opportunity cost of delaying foraging by engaging in other activities is greater than when foraging in a reward-poor area (Niv et al., 2007).

Thus, expressed in terms of the total reward received, and letting the average rate of reward *available after the delay* be signified by ρ, then at indifference:

(10) $$\begin{array}{r} R\;=\;r\;+\;\rho d \end{array}$$

Thus:

(11) $$\begin{array}{r} r=R\;-\;\rho d \end{array}$$

This arrangement allows for the possibility that a delayed reward carries negative value, whereby a decision-maker would willing to pay so as to be able to resume seeking rewards at the average rate, rather than to wait for the delayed reward.

### Uncertainty

#### Probability and hazard

Whenever reward (capital) is stored for the future, for example when a person lends money to another person or when an animal stores food, there is some possibility that the capital will be lost (for example if a conspecific raids the food store or the debtor defaults on their loan). If there is some constant probability per unit time, referred to as a hazard rate, that future rewards do not materialize as promised, the expected value of reward (magnitude × probability) decreases exponentially with delay and gives rise to exponential discounting (Sozou, 1998).

Following the notation above at indifference:

(12) $$\begin{array}{r} r=Re^{-\lambda t} \end{array}$$

Rearranging:

(13) $$\begin{array}{r} \frac{r}{R}=e^{-\lambda t} \end{array}$$

Where λ denotes a constant hazard rate.

Thus, the agent choosing whether to store reward should adopt a discount rate appropriate to the estimated hazard rate. For example a creditor ought to demand a rate of interest that is commensurate with the risk of the debtor's chance of default per unit time. Interestingly, where the appropriate hazard rate is uncertain, decision-makers ought to weight each possible hazard rate by its probability of being the true rate; such a weighted average of exponential rates approximates hyperbolic discounting (Sozou, 1998; Kurth-Nelson and Redish, 2009). As shown by Sozou (1998), hyperbolic discounting results exactly if:

(14) $$\int_{0}^{\infty }f\left(\lambda \right)e^{-\lambda t}\;d\lambda \;=\;\frac{1}{1\;+\;kt}$$

Where *f*(λ) is a probability density function over hazard rates. The above is satisfied if:

(15) $$\begin{array}{r} f\left(\lambda \right)=\frac{1}{k}e^{-\lambda ∕k} \end{array}$$

i.e., if there is an exponential prior distribution over hazard rates, where *k* determines the shape of this distribution. In support of Sozou's theory, Takahashi et al. (2007) find that the subjective probability of receiving delayed reward in standard intertemporal choice tasks indeed decays hyperbolically.

As the quotation at the start of this article encapsulates, death creates a fundamental motive not to defer rewards for too long. In computational terms death can be considered to be an absorbing state, from which no future reward can be harvested. Notably a hazard rate for the event of dying can be seen to depend on the organism's current state, such that a greater physiological deficit is associated with a greater probability of dying per unit time. The fundamental value of reward is then its effect to reduce the hazard rate for dying (before successfully securing one's legacy). This argument suggests that it is optimal for biological agents to discount future reward more steeply when they are currently far from a physiological set point, based simply on an increased probability of their dying before future reward is attained.

#### Volatility

In summary, environmental hazards create a motive to discount the future, since future rewards might not materialize as promised. In addition, the utility of future rewards might be more uncertain, in the sense of having higher *variance* than immediate rewards (when the variance is known the resulting uncertainty is referred to as risk). Many behavioral economic studies have shown that people tend to be risk averse (Kahneman and Tversky, 1979; Holt and Laury, 2002; Trepel et al., 2005; Andersen et al., 2008; Platt and Huettel, 2008; Jones and Rachlin, 2009), in so far as they will accept a smaller expected payoff over a larger expected payoff with higher variance. If future events tend to evolve with a random component, the uncertainty associated with future events increases with delay (Mathys et al., 2011). To take an example, a decision-maker responding to a discounting questionnaire might have some degree of uncertainty about the subjective utility of a $20 payout received immediately (if this appears implausible, imagine being paid in a foreign currency, whose worth is uncertain). However, owing to volatility governing future events in their lives (e.g., becoming ill, falling into debt, national economic collapse), uncertainty regarding the utility of the $20 ought to increase as it is delayed. In combination with risk aversion this motivates delay discounting. In support of this idea, individual discount rates are correlated with risk aversion (Leigh, 1986; Anderhub et al., 2001; Eckel et al., 2005; Jones and Rachlin, 2009; Dohmen et al., 2010).

Notably, risk aversion can be expressed in terms of *probability* discounting, which is found to be hyperbolic in the odds against receiving a reward. Whilst probability discounting and temporal discounting are often found to be correlated across individuals (e.g., Jones and Rachlin, 2009), they are subject to distinct influences. For example, increasing reward magnitude increases probability discounting (i.e., risk aversion) and decreases temporal discounting (Green and Myerson, 2004). This is often taken as evidence that temporal discounting does not encompass an estimate of the risk associated with future rewards. However, pertinent to discounting is how a person estimates risk to be dependent on delay. Probability discounting offers a measure of risk aversion but does not access this time-dependent representation of risk. In support of this idea Takahashi et al. (2007) find that while probability and temporal discounting are uncorrelated across individuals, temporal discounting does correlate with the rate of decay in the subjective probability of receiving reward after increasing delay. This may help explain why psychiatric disorders are often associated with increased inter-temporal discounting but not necessarily with excessive probability discounting.

## Marr's algorithmic level: processes sub-serving intertemporal choice

In the preceding analysis we have outlined some generic scenarios under which behavior consistent with discounting would be optimal. These scenarios illustrate that discounting need not be considered as a unitary process, rather as (implicitly or explicitly) reflecting an expectation of different environmental contingencies. Under reinforcement learning formulations, such contingencies are seen as engendering transtitions in a state-space (Sutton and Barto, 1998; Dayan and Balleine, 2002; Dayan and Daw, 2008; Kurth-Nelson and Redish, 2009). That it is, an action is assumed to move the agent from one (discrete) state to another, where each state may be associated with a varying quantity of reward. The state-space is equivalent to the vector of rewards described in the classical economic model (Equation 2), though may also be made contingent on the agent's future behavior, giving rise to a matrix, or “decision-tree.” A key question for this account is whether the (discounted) utility of a delayed reward is directly parameterized, which is to say that there is no more inference or learning beyond the state where this utility is considered, or whether the delayed reward is instead considered as part of a cascade of preceding states.

### A parametric discount function?

If higher organisms indeed represent a discount function parametrically, they would require a widespread and efficient system for making this information accessible for decision-making. Neuromodulatory systems, with their diffuse connections to many areas of the brain, would be well placed to achieve this, and several authors have speculated that neuromodulators, such as dopamine and norepinephrine might represent some of the relevant parameters. For example, Niv et al. (2007) have proposed that the average rate of reward is signaled in the mammalian brain by tonic levels of extracellular dopamine in the striatum, suggesting that increased striatal dopamine availability might increase discounting by increasing the implicit opportunity cost of delay. Commensurate with this hypothesis, systemic administration in humans of the dopamine precursor l-Dopa increases discount rates (Pine et al., 2010), although potentially countervailing evidence is that decreasing dopamine transmission in rats by administration of haloperidol (Denk et al., 2005) or flupethixol (Floresco et al., 2008) has been found to increase discounting, or in other studies to exert no significant effect on discounting (Winstanley et al., 2005).

Similarly, a good deal of decision-making neuroscience seeks to uncover how uncertainty is represented neurally (see Behrens et al., 2007; Wilson et al., 2010; Mathys et al., 2011; Nassar et al., 2012). A recent suggestion is that operating in an unstable environment is associated with tonic release (over a time course of minutes) of norepinephrine (Yu and Dayan, 2003, 2005). The latter would suggest that tonic norepinephrine might signal environmental volatility, and thus influence discounting. Clearly, further psychopharmacological work is needed to fully uncover the role of monoaminergic signaling on discounting behavior. Also, if organisms indeed have a parametric model of discounting in the strictest sense, then this ought be revealed in the manner in which estimates of discounting are updated in light of changes in the environment, and careful behavioral work is required to probe this possibility.

### Discounting as a revealed phenomenon

According to a second possibility outlined above, choosing a delayed reward leads to a cascade of states, and may (or may not) lead to the promised reward, which if it occurs, may be delivered in a variety of future states (just in time for Christmas, after I've been killed by a bus, etc.) (see Peters and Büchel, 2010). If an agent uses this cascade of states to evaluate their actions, only the resulting transitions will endow this action with whatever value percolates through from the end states. Here discounting takes place due to learning and/or inference, where the value of the reward gradually evaporates as inference (or learning) propagates through a cascade of states. Given the properties of organisms and their environments, as outlined above, behavior consistent with discounting would simply emerge as the end result of applying these learning processes to situations where there is delay in the receipt of reward. Under this possibility, in terms of the economic model, all relevant information is summarized in an agent's utility function, which then implicitly incorporates the discount function. It appears likely that organisms use parallel mechanisms to calculate the value of the resulting state-space, operating across different timescales of information integration, ranging from updating innate behaviors through evolution, through learning from experience, to inferring future states via deployment of a cognitive map or model of the world.

Reliable valuations may be refined and passed on through genetic inheritance and evolution. For example, the possibility of death, and its associated opportunity cost, is likely incorporated through evolution, whereby internal states deviating from a homeostatic ideal, such as hunger and thirst, are assigned an innate cost as a proxy (see Keramati and Gutkin, 2011). Thus, discounting for food would be expected to increase when hungry, due to innate negative value associated with prolonging a state of hunger. Furthermore, actions themselves might in some cases be selected from an innately determined repertoire. Through Pavlovian conditioning, a stimulus (termed unconditioned stimulus, US, e.g., food) that elicits an innate response (the unconditioned response, e.g., salivation), can become associated with another stimulus (conditioned stimulus, CS, e.g., a tone), such that the latter subsequently becomes capable of eliciting an appropriate innate response independently (Rescorla and Solomon, 1967; Williams and Williams, 1969; Hershberger, 1986; Pavlov, 2003). Here the conditioning process, whereby CS becomes associated with US, can incorporate the cost of delay to conform to the optimal adaptations of some of the computational processes above. For example, if delivery of food follows a tone, with an intervening delay of 10 s, the “Pavlovian value” of the tone may be temporally discounted by a given proportion per unit time relative to that of the food (Domjan, 2003). Algorithmic accounts of classical conditioning, such as temporal difference learning, thus incorporate an exponential discount factor (O'Doherty et al., 2003; Moutoussis et al., 2008; Dayan, 2009; Kurth-Nelson and Redish, 2009). Exactly how such discounting is represented at a neurobiological process level remains unclear, but the influences outlined must be important. For example, the incremental process of temporal-difference learning, including Rescorla-Wagner learning (Domjan, 2003), means that the strength of the association between CS and US comes to reflect their probabilistic relationship.

Organisms can also learn the value of actions based simply on whether or not they yielded benefits in the past, referred to as instrumental conditioning (Domjan, 2003). In algorithmic terms, this can be most parsimoniously achieved by integrating the history of reinforcement following a given action, without representing an explicit model of the relationship between actions and their outcomes (Watkins and Dayan, 1992; Daw et al., 2005; Seymour et al., 2005; Schultz, 2006; Moutoussis et al., 2008; McDannald et al., 2011). This is referred to as *model-free* reinforcement learning, and corresponds to the “Thorndikian” Law of Effect (Thorndike, 1927), or “habit” learning (Dickinson et al., 1995; Ouellette, 1998; Neal, 2006; Tricomi et al., 2009; Dolan and Dayan, 2013; Orbell and Verplanken, 2014). Instrumental learning would be expected to incorporate discounting, to the extent that the environmental influences described earlier in this article affect the timecourse of reward contingent on a particular action.

Finally, biological agents can be availed of a cognitive map, or model, of the world, detailing the results of different actions and their respective values (Dickinson and Balleine, 1994; Balleine and Dickinson, 1998; Gläscher et al., 2010; Daw et al., 2011; McDannald et al., 2011). The choice of action proceeds by thinking forward through the map (or tree), and considering the consequences of alternative actions (see Seymour and Dolan, 2008). This mode of control is referred to in reinforcement learning applications as *model-based* (Gläscher et al., 2010; Daw et al., 2011; Wunderlich et al., 2012; Smittenaar et al., 2013; Lucantonio et al., 2014), and corresponds to the definition of goal-directed behavior in animal learning as being rapidly sensitive to changes in the contingency between action and outcome, or to devaluing the outcome (Dickinson and Balleine, 1994; Balleine and Dickinson, 1998). An advantage of the model-based approach lies in its flexibility. For example, this approach is necessary to generate appropriate intertemporal choices in esoteric scenarios, to which a smooth discount function is not well adapted. For example, say a generous experimenter offers me a choice between $100 today and $125 4 weeks from today. The knowledge that I will be receiving my monthly pay of $1000 exactly 4 weeks from today, and that without additional income I am likely to exceed my overdraft limit next week by around $50, incurring a heavy fine, would likely encourage me to choose the immediate money. If I were to try choose between the immediate and delayed money according to a parametric discount function *alone*, without considering extraneous sources of (dis)utility, I might lose out to the overdraft fine. In summary, through the above innate and instrumental learning processes, given appropriate experience of the cost of delay, an organism can behave in a manner consistent with discounting without directly computing discounted value at all.

## (Mal)adaptive discounting in psychiatric disorders

We propose that whether parametric, or revealed through the above valuation processes, discounting nevertheless represents encoding of different environmental contingencies. It is therefore noteworthy, where changes in discounting are observed, for example in psychiatric disorders, to consider such changes in light of the environment to which a given individual might be “tuned to” (see also Del Giudice, 2014). The key point here is that, the decision-maker brings to a laboratory intertemporal choice task their previous experience of delay and may also consider the rewards of the task in the context of other future outcomes they expect to receive. We consider particular instances of this below.

### Mania as a state of increased opportunity cost

Might steeper discounting in some pathological states reflect increased estimates of opportunity cost? In support of discounting being sensitive to changes in opportunity cost, discount rates for money have been shown to increase in line with increases in inflation (Ostaszewski et al., 1998). More speculatively, steeper discount rates in childhood and adolescence which decline into adulthood (Green et al., 1999; Chao et al., 2009; Steinberg et al., 2009) might even reflect greater potential for growth in adolescence. We propose that the pathological state of mania is associated with perceived high rates of reward and high growth potential, creating a heightened opportunity cost associated with inaction. Mania is known to be associated with impulsive behavior, such as overspending, rash financial decision-making or drug–taking (Swann, 2009), and one study (Mason et al., 2012) finds evidence for steeper discounting in an intertemporal choice task with real-time delays in the order of seconds in individuals prone to hypomanic symptoms.

Notably growth potential creates something of a paradox. On the one hand investing reward to achieve growth implies that the decision maker has adopted a long-term view. On the other hand, having something worthwhile to invest in favors choices that obtain rewards sooner rather than later, so that they too can be invested. For example, imagine you are starting a new business venture. Whilst this is necessarily a long-term project, you might sacrifice other potential rewards, such as your health or relationships, in order to invest resources in the business, which can be seen as borrowing predicated on a high level of return from your new business. Manic individuals generate novel, and often unrealistically ambitious, goals, for example, enlisting on education courses, or indeed starting new business ventures (DSM V, 2013). We propose that these goals create high opportunity costs to delaying reward, increasing preference for immediate rewards, so as to enlist resources for goal-pursuit. This offers a putative psychological explanation for why increased impulsivity in mania (Swann, 2009), including steeper discounting (Mason et al., 2012), manifests alongside an apparent increase in goal-directed activity.

The investment in apparently long-term goals in mania seems to occur at the expense of patients correctly “playing out” or “forward modeling” future scenarios themselves. This explains why the same (mal)adaptation is found across several behavioral domains. McClure and colleagues (McClure et al., 2004, 2007) have suggested that the explicit influence of larger-later options on behavior is associated with greater cognitive control, which is reduced in mania in tandem with prefrontal activation (Murphy et al., 1999; Townsend et al., 2010). This reduction in “forward modeling” is in fact consistent—if not necessary—for the suggestion we make here to work. That is, if a person with mania were to consider in detail the path ahead leading to their goals, they would realize that the projection implicit in their growth estimate is unrealistic and they would feel able to afford to be patient. A further interesting possibility, discussed further below, is that such forward modeling itself takes time, and that in the face of high opportunity costs, the depth of such model-based strategies is reduced in favor of more rough-and-ready heuristics, or more Pavlovian or habitual responding (Dezfouli, 2009; Huys et al., 2012). Future investigations of mania might focus on measuring beliefs about growth and opportunity cost directly, and whether such beliefs correlate with changes in discounting. Interestingly, Dezfouli (2009) similarly propose that the abnormally high rewards engendered by drugs of abuse lead to an artifically elevated estimate of the average reward rate in the environment, and that this accounts for increased discounting seen amongst substance abusers (e.g., Kirby et al., 1999; Kollins, 2003; Kirby and Petry, 2004).

Finally, we have shown above how an increase in the rate of reward available from activities *other than those currently on offer* increases impatience to complete the current activity as soon as possible (i.e., increases discounting for rewards obtained from the task in hand). Niv et al. (2007) use the same approach to explain variations in response vigor. In their model they propose that the agent can choose to reduce latency of its responses, at some energetic cost that is proportional to the latency reduction. Thus, choosing how *quickly* to perform a particular action itself becomes an intertemporal choice. As their model illustrates, greater vigor (shorter response latency) is then optimal where the average reward rate is higher, in order that agents can resume reward seeking as soon as possible. This description accords well with that of mania, where sufferers often describe the need to complete various tasks with great urgency and where the general vigor of behavior is markedly increased. Furthermore, the model of Niv and colleagues incorporates a latency-independent cost associated with switching tasks. As the authors show, at high reward rates latency-dependent costs tend to dwarf the switching cost, leading to greater task switching than at low reward rates. This too is in keeping with behavior exhibited in manic states, where sufferers have difficulty sustaining tasks.

### Economic poverty as a deficit state

In keeping with the normative notion that deficit states increase a hazard rate for losing out on future reward, discounting indeed tends to be higher in states of monetary or physiological deficit. For example, steeper discounting is observed in individuals with lower incomes (Green et al., 1996; Reimers et al., 2009), an effect which remains after controlling for level of education. Of course, such studies are correlational, making it difficult to conclude that changes in income directly alter discounting. However, an interesting study by Callan et al. (2011) provides indirect support for a more causal role of low income in increasing discounting. The authors found that a manipulation which lead people to believe that their income was lower than their peers brought about an increase in discounting, relative to a group who were lead to believe that their income was similar to that of their peers. The manipulation was interpreted as priming personal notions of deservedness, though this might just as easily be formalized as a shift toward a perceived deficit state. In a conceptually related study Haushofer et al. (2013) performed an experiment in which subjects performed an effort task for monetary reward, after which different groups received either an increase in income from a low starting endowment, or a decrease in income from a high starting endowment. The design thus allowed the effect of (experimental) wealth changes to be dissociated from absolute wealth. Subjects' temporal discount rates were measured before and after the task, with the finding that negative income shocks lead to an increase in discounting, while positive income shocks effected a small decrease in discounting. Starting wealth was found to be unrelated to discounting. Notably, the size of an experimental endowment might not be expected to have an effect on discounting, since the endowment was likely to be small in comparison to subjects' total real-world wealth. The effect of negative income shocks, which might be interpreted as having primed an increased hazard rate for future earnings, suggests that instability in earnings, rather than simply total wealth, is an important determinant of the relationship between socioeconomic status and discounting.

A study in women deprived of food and water (for 4 h after their usual waking time) found that women given a pre-loading meal prior to testing chose an option leading to the delayed, rather than immediate, delivery of juice significantly and significantly more so than women who had not received a preloading meal (Kirk and Logue, 1997). Also, Wang and Dvorak (2010) measured monetary discounting before and after participants drank either a sugary or a sugar-free drink (both caffeine-free), finding a significant decrease in discounting in the group who drank the sugary drink and a significant increase in the control group. This finding suggests that raising blood glucose decreases discounting, an idea congruent with increased discounting associated with deficit states.

Economic poverty may well underlie *some* of the steeper discounting seen in psychiatric disorders, through an association between mental illness and lower socioeconomic status (e.g., Weich and Lewis, 1998; Lorant et al., 2007) (however in several studies associations remain after controlling for socioeconomic characterisitics). Notably, there may be an interdependent relationship between low socioeconomic status, discounting, and mental ill health, whereby impatience for rewards leads to maladaptive choices such as substance misuse, which in turn are associated with worsening finances, further increases in discounting and increased risk of psychiatric disorder (e.g., Fields et al., 2009b; Leitão et al., 2013). A similar idea has been championed by Bickel et al. (2014b), who propose that the environment associated with low socioeconomic status promotes steeper discounting, which in turn engenders unhealthy choices, thus contributing to known socioeconomic gradients in health status (Adler and Rehkopf, 2008). This is supported by evidence that cigarette smoking, obesity, alcohol use and illicit drug use all exhibit negative relationships with socioeconomic status (Conner and Norman, 2005), that these behaviors are associated with poor executive functioning (e.g., Bickel et al., 2012a), and that economic poverty is prospectively associated with poor executive functioning (Lupien et al., 2007; Noble et al., 2007; Evans and Schamberg, 2009). We discuss this interaction between environment and cognition in Section The Cost of Thinking in Economic Poverty, Borderline Personality Disorder and Schizophrenia below.

### ADHD as a deficit state

Interestingly, the effects of deprivation appear to cross modalities of reward. For example, mild opioid deprivation in opioid dependent individuals increases discounting for money as well as heroin (Giordano et al., 2002). Arguably this might be motivated by a desire on the part of subjects to obtain money sooner so as to buy drugs. However, it might equally be attributable to a more global alteration in decision-making associated with physiological deficit states (see also Loewenstein, 1996; Metcalfe and Mischel, 1999). In further support of this idea, exposure to erotic cues increases discounting for money, as well as for candy bars or soda drinks in men (Van den Bergh et al., 2008). Furthermore, the effect of sex cues to increase discounting for food and drink rewards was attenuated by satiation with money, providing evidence for a global physiological signaling mechanism. Niv et al. (2007) propose that this mechanism “global drive” mechanism might involve modulation in tonic dopamine signaling.

In some cases steeper discounting observed in psychiatric disorders might reflect processes associated with normal deficit states. ADHD is a possible example. ADHD is defined by behavioral symptoms of inattentiveness, over-activity and impulsivity, of long-standing duration and is most commonly diagnosed in school-aged children (DSM V, 2013). Many studies have shown that children with ADHD have a greater tendency than controls to choose immediate over delayed rewards in single choices (e.g., Sonuga−Barke et al., 1992; Schweitzer and Sulzer−Azaroff, 1995; Kuntsi et al., 2001; Bitsakou et al., 2009; for reviews see Luman et al., 2005; Paloyelis et al., 2009) and (relative to controls) are biased toward choosing tasks which yield earlier, rather than delayed, reinforcement (Tripp and Alsop, 2001). Also, on delay of gratification tasks (Mischel et al., 1989) children with hyperactivity exhibit a greater tendency to terminate the delay to obtain a smaller reward, rather than waiting an allotted time for a larger reward (Rapport et al., 1986). Furthermore, several studies now report steeper monetary discounting in children with ADHD (Paloyelis et al., 2009; Scheres et al., 2010; Wilson et al., 2011; Demurie et al., 2012) or in adults with previous ADHD (Hurst et al., 2011).

We hypothesize that the increased discounting rates found in ADHD reflect both the well-known genetic vulnerability for this disorder but also encode the more deprived environments that lead to increased expression of this disorder (Apperley and Mittal, 2013; Russell et al., 2015). In support of this, in one study boys with ADHD symptoms who had been reared in deprived institutions showed increased aversion to delay compared with ADHD controls compared to less deprived patients (Loman, 2012). Thus, seeking of immediate reward in ADHD might reflect underlying mechanisms linking increased discounting with states of internal deprivation. One such mechanism would be that outlined above of higher rates of reward available from alternative tasks. For example, say that children with ADHD have an internal state resembling a deprivation of loving attention; their performance of tasks that do not offer this attention, such as quiet private study, is likely to be more impatient, so as to more quickly return to actions that do command attention from others.

### Increased estimates of uncertainty and hazard

Although conventional discounting tasks offer choices between rewards that are promised to be delivered with certainty, decision-makers likely come to the task with a prior belief regarding the level of hazard in the environment, and so tend to implicitly distrust the experimenter's assertion that the future rewards are guaranteed. In support of this, discount rates amongst cigarette smokers have been shown to correlate positively with their belief that the future reward will be delivered (Reynolds et al., 2007). Also, within a standard discounting questionnaire, people discount more steeply when rewards are framed as being received from fictive characters rated as untrustworthy, as opposed to from characters perceived as trustworthy (Michaelson et al., 2013).

In an interesting study, Callan et al. (2009) measured discounting in 56 undergraduate students who first watched an interview with a HIV-positive woman. One group were told that she had acquired HIV through unprotected sex and the other group that she had acquired the virus via an infected blood transfusion. The latter group exhibited significantly steeper discounting, an effect which was proposed to result from the story of the infected blood transfusion having primed a belief that the world is unjust. A related explanation, independent of feelings of injustice *per se*, would be that the transfusion scenario increased the perceived hazard rate for adverse life events.

Finally, as described previously, the ultimate hazard is that one will die before the future reward occurs. In keeping with this, in a South African population, discounting was found to be higher amongst individuals with the lowest perceived survival probability than amongst those with average survival probability (Chao et al., 2009), and to correlate with the number of bereavements of close family members reported by North Americans (a factor putatively increasing perceived mortality risk) (Pepper and Nettle, 2013). Furthermore, discounting has been shown to increase on conscription into the Israeli army (Lahav et al., 2011), and to be higher in youths living in slums in Rio De Janeiro than in an age matched sample of university students (Ramos et al., 2013).

Populations with psychiatric disorders might well believe that future rewards are less likely to materialize (a higher hazard rate) than do healthy control populations, for quite rational reasons, given their life experiences (Hill et al., 2008). In other words, the past is the best predictor of the future, and this may be why psychiatric disorders associated with hazardous development are characterized by higher discounting rates. Populations with psychiatric illness have experienced an excess of major life events compared with the healthy population (Paykel, 1978), and have excess mortality from physical health conditions compared with the general population (Robson and Gray, 2007). The latter would be expected to be associated with lower perceived survival probability, given correlations between perceived and actual mortality in the general population (Idler and Benyamini, 1997). To our knowledge no previous studies have examined this. This may in turn result in decisions that perpetuate or worsen the disorder. Indeed, Sonuga-Barke has hypothesized that the high discounting rates measured in the laboratory in youths with conduct disorder represent an accurate—and hence adaptive in their native environment—summary of the increased hazards that these youths so commonly have experienced (Barke, 2014). An interesting possibility for future research would be to elicit beliefs of groups with psychiatric disorder about the likelihood that future reward will be forthcoming, and to regress this against their discounting choices. Similarly further research is needed to examine relationships between an individual's experience of significant life events, their confidence in the future, and their level of temporal discounting.

### The cost of thinking in economic poverty, borderline personality disorder and schizophrenia

It appears that a greater engagement of model-based control, a faculty tightly dependent on working memory, is associated with more future-oriented responses on discounting paradigms. Promoting mental simulations of future outcomes by cueing participants with episodes in their lives corresponding to the timing of the options decreases measured discount rates (Peters and Büchel, 2010). Higher working memory capacity is associated with both lower discounting (Shamosh et al., 2008), and an increased emphasis on model-based control (Eppinger et al., 2013), while working memory training in substance misusers has been found to decrease their delay discounting (Bickel et al., 2011b).

In keeping with the above, functional neuroimaging studies have found that the dorsolateral prefrontal cortex (dlPFC), an area often implicated in tasks dependent on working memory (Curtis and D'Esposito, 2003), is sensitive to model-based learning signals (Gläscher et al., 2010). This area is also known to be active when choosing delayed rewards on intertemporal choice paradigm (McClure et al., 2004, 2007). Furthermore, disrupting dlPFC function (using either transcranial magnetic stimulation or transcranial direct current stimulation) both decreases the emphasis on model-based control (Smittenaar et al., 2013) and increases temporal discounting (Hecht et al., 2013). The process of mentally simulating future outcomes is also known to be dependent on the hippocampus (Hassabis et al., 2007; Johnson et al., 2007; Schacter et al., 2008; Schacter and Schacter, 2008), and rats with hippocampal lesions have been found to exhibit increased discounting (Mariano et al., 2009). Taken together these results suggest that mental simulation of the future tends to generate more patient intertemporal choices, and that this process is working memory dependent.

A plausible explanation for the above is that mentally simulating the future resolves uncertainty about the utility of larger-later rewards (see Daw et al., 2005). For example, I might be uncertain about how much I am likely to require money in 7 months' time, but if I remember that my partner's birthday is in seven and a half months' time, and I anticipate needing the money to buy him or her an expensive present, I might revise my estimate of the utility of the future money. An interesting possibility is that decision-makers face a trade-off between making the best possible decisions and doing so in a timely manner with the minimum of effort. Model-based simulation of the future is compuationally costly, i.e., consumes time and energy. If conditions are sufficiently unpredictable, then attempting to explicitly plan out future possibilities is futile, and may even be disadvantageous (see Daw et al., 2005). Thus, prolonged exposure to an unstable environment during development ought to both discourage the use of model-based strategies and increase discounting via greater uncertainty associated with future rewards. This possibility would conceptually bind together an unstable childhood environment, diminished cognitive ability and steeper discounting of reward, providing a tentative theoretical basis for explaining the association between these factors in several psychiatric disorders. For example, people with borderline personality disorder are likely to have experienced childhood abuse (Lewis and Christopher, 1989; Ogata et al., 1990; Zanarini et al., 1997), exhibit below average cognitive function (Swirsky-Sacchetti et al., 1993) and discount the future more steeply than healthy controls (Lawrence et al., 2010).

A similar interaction might in part underlie associations between low socioeconomic status, steeper discounting and psychiatric disorder. Bickel et al. (2014a, 2011a) propose a neuropsychological explanation for relationships between low socioeconomic status and unhealthy lifestyle choices, in terms of a dual-systems model of cognition, whereby low socioeconomic status encourages engagement of a more “impulsive” decision-making system, putatively mediated by limbic brain structures, over an “executive” decision-making system, mediated by parts of frontal cortex. The authors point to evidence that several neurocognitive abilities including working memory, declarative memory, and cognitive control exhibit socioeconomic gradients (Noble et al., 2007). This association appears to hold in prospective analyses too. On a developmental timescale, Evans and Schamberg (2009) show that childhood poverty predicts lower working memory in young adulthood, and that high levels of childhood stress mediate this relationship. State-based effects of poverty on cognitive function are also evident, for example Indian sugar-cane farmers exhibit worse cognitive performance before their harvest, when they are poor, than after their harvest, when they are richer, even controlling for levels of stress (Mani et al., 2013). The dual-systems approach is not incompatible with our three-way division of behavioral control. The model-based system for instance appears to depend on executive functions such as working memory, but has the advantage of carrying a specific algorithmic meaning. Also, we envisage the three-controllers as sharing the mutual goal of maximizing reward (Dayan et al., 2006), and suggest that their relative deployment is also subject to a cost-benefit trade-off (Daw et al., 2005; Dezfouli, 2009; Huys et al., 2012). We therefore go as far as to propose that diminished deployment of model-based control in states of deprivation might reflect an evolutionary milieu in which such changes were approximately optimal, for example in response to irreducible future uncertainty.

Deficits in future thinking appear likely to underlie steeper discounting seen in patients diagnosed with schizophrenia compared with healthy controls (Heerey et al., 2007, 2011), in keeping with observations that such patients often exhibit cognitive and executive dysfunction. Furthermore, patients with schizophrenia exhibit atrophy of frontal and temporal brain regions (Madsen et al., 1999; Velakoulis et al., 2001; van Haren et al., 2008), a pattern which would be expected to be accompanied by shortened time perspective, given the role of these structures in imagining future scenarios (Hassabis et al., 2007; Johnson et al., 2007; Schacter et al., 2008; Schacter and Schacter, 2008). Heerey et al. (2011) present evidence to support this view, comparing measures of discounting, cognitive function and “future representation” in 39 patients with schizophrenia and 25 healthy control participants. Patients discounted more steeply than controls, and when asked to list events which they thought might happen to them in their lives, on average reported future life-events that were nearer in time. This shortened future perspective correlated with lower working memory scores in both patients and controls, to the extent that controlling for working memory abolished the effect of schizophrenia status on discounting. These results suggest that discounting deficits in schizophrenia are attributable to an impaired ability to imagine the future, a faculty that is limited by working memory capacity.

## Future directions

The above account leaves considerable room for future research. The foregoing discussion has largely focused on appetitive processes evoked in the appraisal of future *rewards*. A complementary, but distinct, set of principles might apply to how humans evaluate future punishment. For example, as a complement to the theory that tonic dopamine signals the average reward rate, it has been proposed that tonic serotonin signals the long run average punishment rate, and thus controls the vigor of avoidance behavior (Dayan, 2012a,b, see also Crockett et al., 2012). This idea might hold relevance for increased discounting in depression, which is associated with both marked avoidance (Ferster, 1973) and possible serotonergic abnormalities (e.g., Mann et al., 2000). Although a normative account of the role of serotonin in depression remains elusive, it is interesting that decreasing serotonin availability (achieved by tryptophan depletion) in healthy subjects acts to increase discounting (Tanaka et al., 2007; Schweighofer et al., 2008), commensurate with increased discounting seen in depression (Takahashi et al., 2008; Dennhardt and Murphy, 2011; Dombrovski et al., 2011, 2012; Imhoff et al., 2014; Pulcu et al., 2014) (For further discussion of temporal preferences for punishment see Berns et al., 2006; Story et al., 2013, 2015).

A further area for future research concerns the effect of stress on discounting (e.g., Diller et al., 2011; Kimura et al., 2013). A recent meta-analysis (Fields et al., 2014) of 16 studies examining the relationships between delay discounting or delay of gratification and subjective or physiological measures of stress and found that stress was associated with steeper discounting, with a large aggregate effect size (Hedge's *g* = 0.59). Seemingly contradicting these findings, low baseline cortisol levels have been associated with *increased* delay discounting (Takahashi, 2004), and similarly predict higher discounting at 6 month follow up (Takahashi et al., 2009). A possible explanation would be that baseline stress and responsivity to stress manipulations exert distinct influences on discounting. In part supporting this idea, Lempert et al. (2012) found that when placed under stressful conditions, individuals with low trait perceived stress showed higher discounting than those with high trait perceived stress, perhaps reflecting greater responsiveness to acute stressors in subjects with low trait stress. In addition acute administration of hydrocortisone, a key hormone involved in stress response, has been found to cause a short-lived increase in discounting (Cornelisse et al., 2013). Further work is required to understand the relationships between baseline and induced stress and their interaction with discounting, as well as to characterize stress in terms of the information content of stressful situations.

The above account has not specifically addressed willpower. Several lines of evidence point to the fact that humans often renege on best-laid plans, in favor of immediate consumption. We propose that this results since people are poor in predicting in advance the effect of conditioned cues and motivational state changes on their behavior (see also Loewenstein, 1996; Metcalfe and Mischel, 1999; Read, 2001; Chapman, 2005; Dayan et al., 2006; Story et al., 2014). Thus, one might plan to abstain from eating dessert as part of a diet plan, but find it harder to resist when presented with a piece of cake (see for example Read and Van Leeuwen, 1998; Allan et al., 2010) and relapses in drug-taking behavior following abstinence commonly occur after exposure to a previous drug-taking environment (O'Brien et al., 1998). Similarly, people appear poor in predicting their behavior in future motivational states that differ from their current motivational state. For example, in a study of analgesic preferences for childbirth (Christensen-Szalanski, 1984), women asked roughly 1 month in advance of labor preferred to avoid invasive spinal anesthesia in favor of less invasive but less effective pain relief methods, however during active labor women frequently reversed preference and opted for anesthesia. “Battles of will” then consist in the attempt to punish or extinguish existing habitual or Pavlovian responses through the imposition of countervailing model-based (goal-directed) valuations. Hyperbolic discounting theoretically gives rise to similar intertemporal choice conflicts, but considered alone has difficulty accounting for the state-dependence of real world failures of self-control. Thus, in the study of Christensen-Szalanski (1984) it seems likely to be the transition into a painful state that brings about a shift in womens' preferences for analgesia, rather than the time preceding childbirth *per se* as hyperbolic discounting would suggest. An interesting direction for future research will be to examine whether individuals with psychiatric disorders, for example borderline personality disorder, exhibit greater choice inconsistency over time, relative to controls. This possibility would accord with a well-esteemed theory that individuals with borderline personality disorder are impaired in modeling mental states (Bateman and Fonagy, 2004).

Another interesting direction not explored here concerns discounting of *past* rewards (Yi et al., 2006; Bickel et al., 2008). Discounting for past rewards has been shown to be systematic and hyperbolic in form, and is correlated with the degree of future discounting across individuals (Yi et al., 2006). Furthermore, cigarette smokers are found to discount past, as well as future, rewards more steeply than non-smokers (Bickel et al., 2008). Symmetry between past and future discounting is in keeping with evidence that remembering the past and imagining the future are both dependent on the hippocampus (Hassabis et al., 2007; Johnson et al., 2007; Schacter et al., 2008; Schacter and Schacter, 2008). Notably past discounting is difficult to directly account for in terms of some of the informational influences suggested in this article. Growth potential for example ought to motivate having received rewards in the distant past, since these should have had time to accrue greater value. Further work is clearly needed to understand the possible normative basis of past discounting. One possibility is that factors tending to foreshorten model-based consideration of future outcomes, such as uncertainty, also dimish retrieval of episodic memories, leading to a narrowing of temporal perspective. Notably, the learning rate in model-free reinforcement learning algorithms corresponds to an exponential discount factor for past reward. Yechiam et al. (2005) have shown that susbtance misusers and inidividuals with ventral medial prefrontal cortex lesions both exhibit increased learning rates on the Iowa gambling task, where an excessive focus on recent reinforcement is disadvantageous. This suggests that high learning rates might reflect a form of “retrospective impulsivity,” through assigning too little weight to distant past experience. Further work is required to explore this possibility.

A final consideration is that of how discounting differs between different forms of outcome. Discounting for several forms of appetitive outcome shows consistency across individuals, for example discount rates for money are strongly and significantly correlated with other forms of appetitive outcome, such as the discounting of cigarettes for cigarette smokers, the discounting of heroin for opioid-dependent outpatients and the discounting of food amongst college students (Odum, 2011; Pearson *r* = 0.93; *p* = 0.0007 for money vs. the mean of all other outcomes). However, rates are not *identical* across commodities: people tend to discount primary reinforcers such as food, water and sex more steeply than money (Lawyer et al., 2010; Odum, 2011; Jarmolowicz et al., 2013) and a number of studies have shown that people with substance dependence discount their drug of abuse more steeply than money (e.g., Madden et al., 1997; Bickel et al., 1999; Petry, 2001). Steeper discounting for primary reinforcers might reflect their greater engagement of innate appetitive systems. In other words, deliberative consideration of primary reinforcers might increase attention to the relevant underlying deficit state (drive). Steeper discounting then putatively results due to the negative Pavlovian value associated with prolonging the deficit state. Further research is needed to examine this possibility.

Interesting results have been obtained when discounting choices are made across different commodities, for example in choices between money now vs. cigarettes later, termed cross-commodity discounting (CCD), as opposed to single-commodity discounting (SCD). For instance, Bickel et al. (2011a, 2007) examined discounting in cocaine-dependent individuals between cocaine now vs. cocaine later (C-C), money now vs. money later (M-M), cocaine now vs. money later (C-M), and money now vs. cocaine later (M-C) conditions, where the amounts of money and cocaine across conditions were equated in immediate worth. Consistent with previous findings, C-C discount rates were significantly greater than M-M discount rates; indeed there was a significant main effect of changing the delayed commodity to cocaine, consistent with cocaine being discounted more steeply than money. However, the authors found that, whilst C-M and M-M discounting were statistically indistinguishable, M-C discount rates were significantly higher than C-C discount rates. Wesley et al. (2014) broadly replicate this result, and Jarmolowicz et al. (2014) find a similar pattern of findings for money vs. sex CCD, wherein a M-S condition was associated with the steepest discounting. A possible explanation in terms of the classical economic model would be that cocaine (or sex) is both discounted more steeply and has a *less* concave utility function than money. Bickel et al. (2011a, 2007) illustrate this possibility though favor an explanation in terms of a framing effect. We propose a framing hypothesis whereby primary reinforcers are associated with a steeper implicit hazard rate than money (this might in part underlie their steeper discounting, but is of itself insufficient to explain the above findings); SCD then hypothetically diminishes the implicit hazard rate, by priming the idea that the commodity will definitely be received sooner-or-later. By contrast, the implicit exchange of money for primary reinforcement in CCD hypothetically amplifies the hazard rate for the delayed commodity, by priming the notion that the delayed commodity is not guaranteed. This hypothesis leads to the observed interaction, with the steepest discounting for CCD in which primary reinforcement is delayed, and is an eminently testable. The possible modulation of such cross-commodity effects in various psychiatric disorders might offer further clues as to the underlying decision mechanisms at play.

In summary we have reviewed motivations for steeper discounting of delayed reward. Discounting tends to be increased across a broad range of disorders, including ADHD, schizophrenia, bipolar disorder, hypomania, depression, borderline personality disorder and substance misuse disorders. We have proposed that these findings can be parsimoniously understood by examining the *reasons* why people should discount the future, namely the opportunity costs of delay, uncertainty associated with future outcomes and the cognitive costs of resolving this uncertainty. We have detailed different types of *information processing* in the brain that can take these factors into account, broadly distinguishing “parametric discounting,” whereby rewards labeled as delayed are automatically discounted as a function of delay, vs. “planful discounting” where the factors associated with the delay are accounted for in the course of learning. Where possible we have attempted to map these normative influences onto putative, albeit broad neurobiological *mechanisms*. More generally we propose that this approach, that is, attempting to understand the biological substrates of psychiatric disorder in terms of their physiological *function*, and in light of a person's life history, is key to bridging psychosocial and biological conceptions of mental illness. We accept that our use of this approach here might appear speculative. In essence, we feel is this justified given the emerging nature of the field and await further research developments with eager interest.

## Funding statement

This work was supported by the Wellcome Trust [Ray Dolan Senior Investigator Award 098362/Z/12/Z]. The Wellcome Trust Centre for Neuroimaging is supported by core funding from the Wellcome Trust 091593/Z/10/Z. Dr. Moutoussis is also supported by the UCLH Biomedical Research Council.

### Conflict of interest statement

The authors declare that the research was conducted in the absence of any commercial or financial relationships that could be construed as a potential conflict of interest.
